# Color vision in insects: insights from *Drosophila*

**DOI:** 10.1007/s00359-019-01397-3

**Published:** 2020-02-04

**Authors:** Christopher Schnaitmann, Manuel Pagni, Dierk F. Reiff

**Affiliations:** Department for Animal Physiology and Neurobiology, Institute of Biology I, Albert-Ludwigs-University Freiburg, Freiburg, 79104 Germany

**Keywords:** Rhodopsin, Photoreceptor, Spectral processing, Wavelength discrimination, Color opponency

## Abstract

Color vision is an important sensory capability that enhances the detection of contrast in retinal images. Monochromatic animals exclusively detect temporal and spatial changes in luminance, whereas two or more types of photoreceptors and neuronal circuitries for the comparison of their responses enable animals to differentiate spectral information independent of intensity. Much of what we know about the cellular and physiological mechanisms underlying color vision comes from research on vertebrates including primates. In insects, many important discoveries have been made, but direct insights into the physiology and circuit implementation of color vision are still limited. Recent advances in *Drosophila* systems neuroscience suggest that a complete insect color vision circuitry, from photoreceptors to behavior, including all elements and computations, can be revealed in future. Here, we review fundamental concepts in color vision alongside our current understanding of the neuronal basis of color vision in *Drosophila,* including side views to selected other insects.

## Introduction

In the life of insects and many vertebrates including humans, color vision is abundant and plays a central role in guiding behavior. It allows the discrimination of spectrally distinct stimuli regardless of their relative intensity. In humans, the perception of color is described by the attributes color hue, saturation, and brightness (Kelber and Osorio 2010; Lunau 2014), and it can be associated with additional sensations, emotions, or call up memories, explaining the importance of color for arts (Backhaus et al. 1998). How insects including *Drosophila* perceive color we cannot know. However, we can analyze and quantify the spectral discrimination abilities of insects in behavioral experiments, and we can refer to their color vision and presented color stimuli using the parameters spectral information, spectral purity, and intensity. Analyzing the underlying neuronal circuitries in insects and other taxa can provide us with a better understanding of the question whether the miniature brains of insects employ similar or different physiological, computational, and network mechanisms to derive information on spectral content as the much larger brains of, for instance, vertebrates (Dacey and Packer 2003; Gegenfurtner and Kiper 2003; Kelber et al. 2003; Gegenfurtner 2003; Solomon and Lennie 2007; Osorio and Vorobyev 2008; Jacobs 2008).

**Fig. 1:**
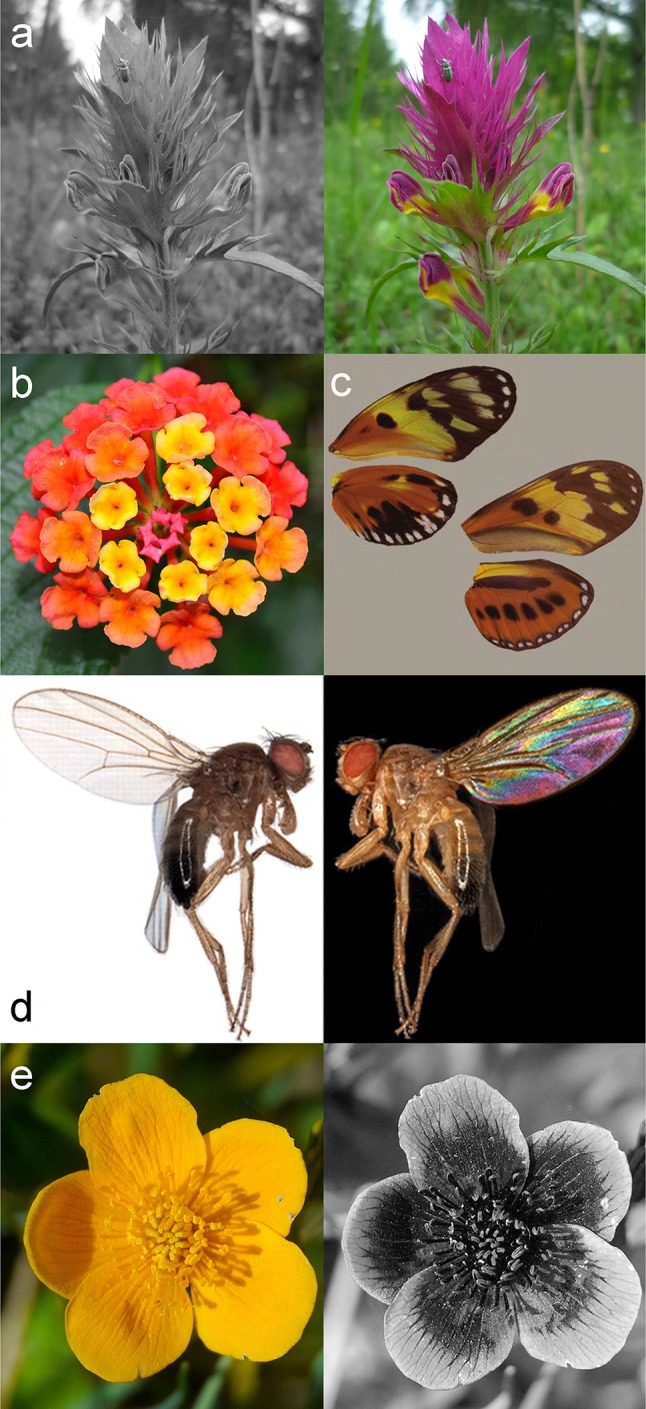
Color vision facilitates image segmentation, object identification, and underlies diverse behaviors. **a** Addition of spectral contrast to the black and white image facilitates the segregation of objects from background. Field cow-wheat (*Melampyrum arvense*) pops out from the meadow when displayed in color. **b** Color vision enables a more accurate judgement of the properties of objects. For instance, floral color change can provide important cues for pollinators. After opening when flowers are still loaded with nectar, the shown *Lantana* (*Lantana camara*) flowers are yellowish. They change to orange and purple-red when nectar is increasingly depleted (Weiss 1991). **c** Color vision can enable intraspecific communication, also in the presence of co-occurring mimics. The wing patterns of *Heliconius* (*Heliconius numata*, upper left) and several closely related genera (*Eueides isabella*, lower right) display a shared warning signal. Yellow pigmentation in *Heliconius numata* with additional reflection in the UV, and additional UV sensitivity are consistent with a trait for intraspecific communication (Bybee et al. 2012). **d** Color vision can enable the detection of wing interference patterns (WIPs) that are displayed by the wings of most Hymenoptera and Diptera (here *Drosophila melanogaster*). WIPs have been suggested to serve intraspecific communication and were recently shown to be an important trait in sexual selection behavior in *Drosophila* (Shevtsova et al. 2011; Hawkes et al. 2019). **e** Insect color vision with sensitivity in the UV range of the spectrum, in addition to sensitivity for longer wavelengths, allows many insects to detect patterns on flowers that are hidden to the human eye. A buttercup flower (*Caltha palustris*) is perceived homogeneous yellow by a human observer (left) although it strongly reflects in the UV range (right, photographed with a 310–390 nm filter and displayed in greyscale). Images in (**c**) modified, Bybee et al. 2012 (**d**) Shevtsova et al. 2011; (**e**) modified, © Dr Schmitt, Weinheim Germany, uvir.eu

The observation that ‘simple organisms’ like insects are capable of color vision has been shown for the first time in the pioneering work of Karl von Frisch (von Frisch 1914). He conditioned honey bees to colored cardboards and showed that trained bees can distinguish the conditioned color from 30 different shades of gray. At this time, color vision was widely considered a privilege of certain vertebrates including humans, despite the many pollinating and frugivorous insects that obviously use spectral content to guide behavior. Today, we know that a wide range of animals from different taxa, among them numerous arthropods, are capable of color vision. Color vision endows these animals with extra power for the generation of contrast that facilitates image segmentation. The blossom of field cow-wheat that is frequently visited by different insects is difficult to recognize in the black and white image in Fig. 1a. However, addition of spectral information to the image makes the flower pop out from the green meadow (Fig. 1a) (colors are named according to human perception throughout the manuscript). Thus, color vision facilitates the identification of objects and enables a better judgement of their quality. The latter is demonstrated, for instance, by the coloration of fruits and flowers that often signal ripeness or that it is worth visiting a plant for its nectar (Fig. 1b). This includes changes in floral color that are exhibited by many angiosperm plants and that have been shown to instruct the behavior of pollinators (Weiss 1991).

Next to this important function in the identification of sources of food, color vision can have an important role in the identification of predators, conspecifics and in communication (Poulton 1890; Osorio and Vorobyev 2008; Lunau 2014; Osorio and Cuthill 2015; Cuthill et al. 2017). For instance, *Heliconius* butterflies display a warning wing pattern that is shared among different local butterfly species. Yellow color patches with additional reflection in the UV range and additional rhodopsin molecules that provide extra sensitivity in the UV range in *Heliconius numata* have been interpreted in the context of intraspecific communication (Kronforst et al. 2006; Bybee et al. 2012) (Fig. 1c). Also, Diptera and Hymenoptera display colorful wing patterns (Fig. 1d; Shevtsova et al. 2011). Two recent studies, one on *Drosophila melanogaster* and one on *Drosophila simulans* demonstrate that these wing interference patterns serve intraspecific communication and sexual selection behavior (Katayama et al. 2014; Hawkes et al. 2019).

The wavelengths contributing to color vision and behavior strongly depend on the types of photoreceptors and their spectral sensitivities. The buttercup flower is uniform yellow for a human observer (Fig. 1e, left). Its UV reflectance (Fig. 1e, right) is not detected by the short (S), middle (M) and long (L) wavelength-sensitive cones that govern color vision in humans, and that have likely evolved to solve other tasks, including the detection of reddish fruits against green foliage (Mollon 1999; Regan et al. 2001; Melin et al. 2013). In contrast, the contained reflection in the UV range is very well visible to many insect pollinators that have photoreceptors with spectral sensitivity in the UV range (Menzel and Backhaus 1991; Briscoe and Chittka 2001; Chen et al. 2013; Lunau 2014). This example highlights that the detection of color depends on who is looking. The spectral properties of photoreceptors and the exact neuronal mechanisms for the comparison of their signals determine the range and type of spectral contrast that can be detected. Thereby, subtle differences in the properties of the color vision system can decide whether or not small differences in the spectral advertising strategies of flourishing plants can be detected, as recently shown for a mixed community of local bee pollinators and their preferred plants (Shrestha et al. 2019).

In local communities of pollinating insects and plants, the wavelengths where sharp changes in the reflectance of flowers occur can correspond well with the wavelengths where pollinators have maximum wavelength discrimination ability, as shown for instance, for different plant–bee communities (Chittka and Menzel 1992; Dyer et al. 2012). In this sender–receiver interplay, it is difficult to judge whether also the visual capabilities of the pollinators influence the floral color display. Using spectral analysis of flower signals and models of insect color vision, this has recently been suggested for a plant–pollinator community with reduced diversity of floral visitors. On a southern ocean island, with flies as the only pollinators, the chromatic capabilities of the fly visual system seemingly impose a filter on floral color display and plant community assembly (Shrestha et al. 2016).

In summary, colored traits and color detection are of great importance in the life of many insects (Hempel de Ibarra et al. 2014; Cuthill et al. 2017; Lebhardt and Desplan 2017). In contrast to bees or butterflies, however, we know relatively little about the ecological functions of color vision in *Drosophila*. Further studies, particularly in its natural habitat that could be related to the behavioral studies under laboratory conditions, would be very valuable (Dickinson 2014).

## Photoreceptor opponency—a hallmark of color vision

**Fig. 2:**
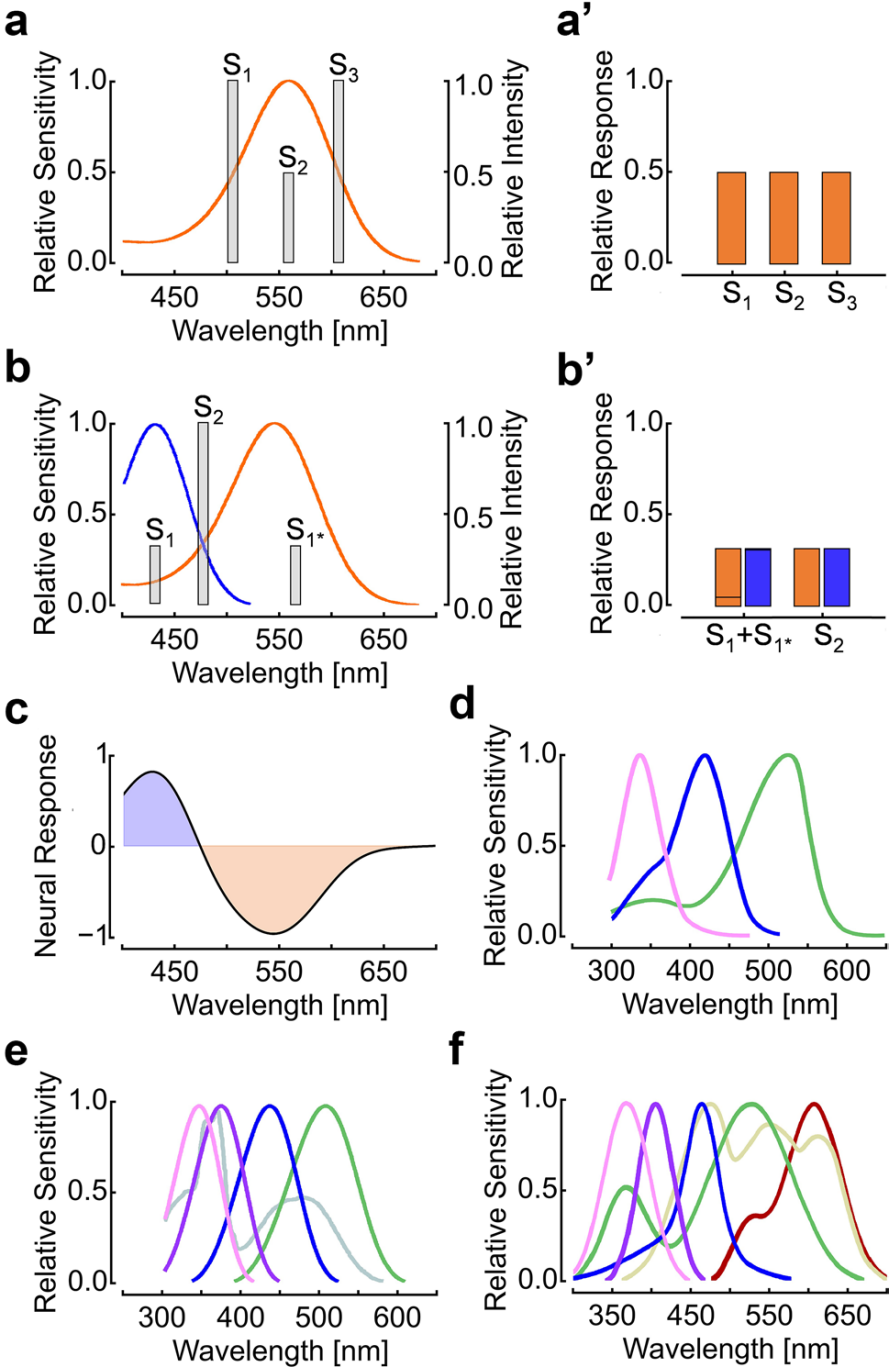
Photoreceptor functions, color opponent processing and photoreceptor sensitivity in selected insects. **a**, **a**′ Principle of univariance: light stimuli S1, S2, and S3 differ in wavelength and intensity (**a**), but elicit identical responses in a given photoreceptor (**a**´). **b** Example for a dichromatic color vision system with short and long wavelength-sensitive photoreceptors. **b**′ The metameric light stimuli (S_1_ + S_1*_) and S_2_ elicit same response in the two types of photoreceptors and are therefore interpreted as same color. **c** Neuronal response of a hypothetic color opponent neuron that receives antagonistic input from the two types of photoreceptors in (**b**). **d** Spectral sensitivities of the three types of photoreceptors in the trichromatic honey bee visual system (peak sensitivity in the UV, blue, and green range of the spectrum). **e** Spectral sensitivities of the five types of rhodopsins expressed in the predominating types of photoreceptors of the *Drosophila* eye (maximum sensitivity at 478 nm (Rh1, gray), 345 nm (Rh3, light purple), 375 nm (Rh4, violet), 437 nm (Rh5, blue), or 508 nm (Rh6, green). An accessory pigment mediates additional UV sensitivity in R1–R6. **f** Spectral sensitivities of the six classes of spectral receptors of *Papilio xuthus*: UV, violet, blue, green (double-green depicted), red and broad-band. Images modified, after (**d**) Osorio and Vorobyev (2008); (**e**) Salcedo et al. (1999) and Schnaitmann et al. (2018), and (**f**) Arikawa (2017)

Color vision is the result of sequential processing stages. In the first stage of color processing, photoreceptors report spectral and intensity changes of light as a change in membrane potential. Hereby, the spectral sensitivity of a given photoreceptor determines its probability to absorb a photon with a certain wavelength. The photoconversion of light energy during phototransduction (revealed in *Drosophila* in great detail and reviewed in Hardie 2001; Hardie and Juusola 2015) is independent of the wavelength of the absorbed photon(s). The information on the wavelength of a photon is lost in the moment it is absorbed by the photoreceptor. An increase in the photoreceptor response can similarly arise from an increase in light intensity or from the wavelength of the stimulus getting closer to the wavelength of maximal sensitivity of the photoreceptor (Fig. 2a). Consequently, a visual system with a single type of photoreceptor cannot discriminate the spectral property of a stimulus from its intensity. Numerous physically different stimuli can elicit same responses (Fig. 2a and a’). These circumstances are the key predictions of the ‘principle of univariance’ (Rushton 1972).

In the second stage of color processing, the responses of different types of photoreceptors with different spectral sensitivities are compared by the nervous system. In this process, two, three or more different types of photoreceptors do per se not overcome the limitations imposed by the principle of univariance. Multiple receptor classes are not sufficient but necessary for color vision. It is the comparison of their activities that provides information about the spectral properties of a visual stimulus (Kelber and Osorio 2010; Mollon 1999). Thus, in its ‘simplest’ form, color vision is dichromatic and employs two types of spectrally distinct photoreceptors (Fig. 2b, b′). Summation of their output yields a measure of absolute intensity, and calculation of the difference between the responses of both types of photoreceptors enables the encoding of spectral information. The resulting color opponent responses have been observed in second- or higher-order postsynaptic neurons that are excited at one wavelength and inhibited at another (Fig. 2c). However, fingerprints of color opponent processing can already be detected in certain photoreceptors and in particular in the presynaptic terminals of photoreceptors (see ‘circuit mechanisms’). In the spectral range where the calculated difference between the photoreceptor sensitivities has its maximum slope, the power of animals to discriminate wavelength differences is often the highest. Still, two different photoreceptor types can be stimulated to the exact same extent by a wide range of metameric stimuli with very different wavelength composition (Fig. 2b, b’). Such metameric stimuli cannot be discriminated by the observer. This ambiguity can be reduced by employing more types of photoreceptors. The number of naturally occurring spectral signals that can elicit identical responses in different photoreceptor types decreased with increasing number of photoreceptors types (Osorio and Vorobyev 2008). The importance of color opponent processing for color vision can hardly be overrated (Chittka et al. 1992; Dacey and Packer 2003; Gegenfurtner and Kiper 2003; Jacobs 2014; Demb and Singer 2015). In fact, the concept of color opponency dominates the way how researchers think about color vision since Hering published his opponent color theory more than 100 years ago. Ever since, the search for individually identifiable neurons that exhibit color opponent processing became an important aspect of color vision research (Hering 1878; Backhaus et al. 1998; Jacobs 2014).

In insects, the diversity of photoreceptors can vary considerably from one species to another. For instance, the visual systems of cockroaches and many ant species have two types of spectrally different photoreceptors (Mote and Goldsmith 1970; Yilmaz et al. 2017). Vision in honey bees involves photoreceptors that are most sensitive in either the UV, blue, or green range of the spectrum (Fig. 2d). In *Drosophila* and a number of other flies, the visual system harbors five photoreceptor types (Fig. 2e). An even larger diversity is found in the butterfly *Papilio xuthus* with a retina containing six ‘classes’ of photoreceptors (Fig. 2f) or the bluebottle butterfly *Graphium sarpedon* that even has 15 types of photoreceptors (Chen et al. 2016).

Does color vision in these animals necessarily involve all photoreceptor types? And does a higher grade of spectral photoreceptor diversity in an animal correlate with better color vision? Experiments comparing trichromatic RGB devices and hyperspectral cameras demonstrate that multiple, narrow-band sensors enable better reconstructions of a given spectrum (Garcia et al. 2015). In biological systems, however, narrow bandwidth photoreceptors come at a cost of a low signal-to-noise ratio (van Hateren 1993; Osorio and Vorobyev 2008). Also, increased diversity of both photoreceptors and neural circuits that mediate their comparison increases the metabolic and other costs of every bit of encoded spectral information (Niven and Laughlin 2008). Thus, the question arises how many types of different photoreceptors with what kind of spectral tuning properties (shape and width) are optimal for color vision. Studies that used spectra of natural scenes with assumed ecological relevance and models of color vision in different animals suggest that the encoding of spectral information is not improved by more than five types of photoreceptors (reviewed in Osorio and Vorobyev 2005). Thus, the function of higher photoreceptor diversity in color vision remains an open question. In some animals, specific photoreceptor types might drive wavelength-specific behavior (Kelber et al. 2003; Kelber and Osorio 2010).

Detailed insights into the diversity of insect retinae and the contribution of photoreceptors to behaviors guided by spectral cues have been revealed (Kelber et al. 2003; Kelber and Osorio 2010; Hempel de Ibarra et al. 2014; Arikawa 2017). However, our understanding of the molecular mechanisms, physiology, and circuit implementation of color vision in insects is still limited. Help might come from *Drosophila* where advances in genetic targeting of single cell types, anatomical, physiological, perturbational, and behavioral investigation open new avenues to address these questions.

## The *Drosophila* retina and optic lobe

**Fig. 3:**
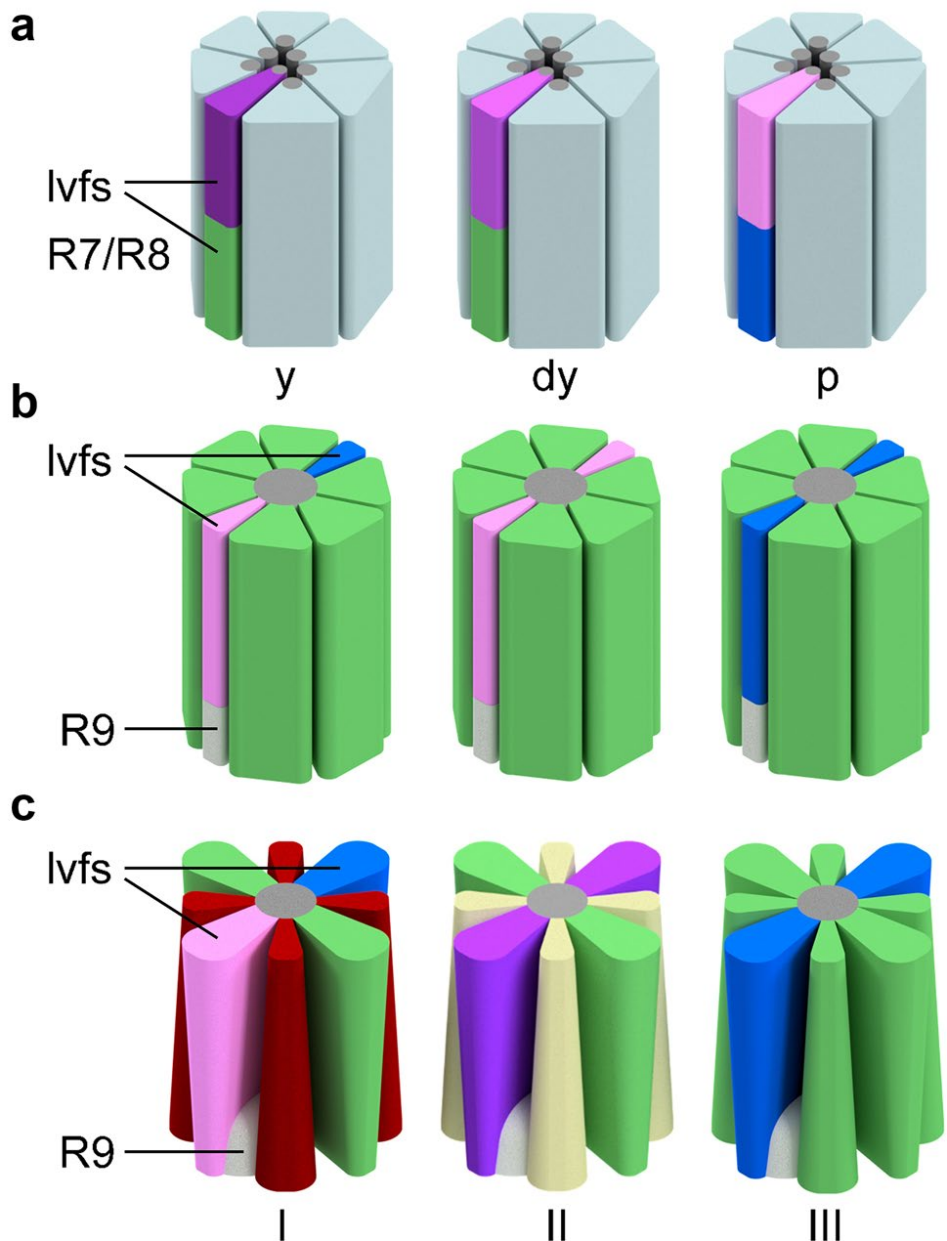
Schematic representation of photoreceptor composition in the predominating types of ommatidia in the *Drosophila*, honey bee, and butterfly (*Papilio xuthus)* eye. **a** In *Drosophila*, rhodopsin expression in the long visual fibers (lvfs) R7/R8 differs in yellow (y), dorsal-yellow (dy, in the dorsal third retina), and pale (p) ommatidia. R7p/R8p express *rh3*/*rh4* (light purple/blue), R7y/R8y express *rh4*/*rh6* (dark purple/green), and dy R8/R7 express *rh6* and *rh3* + *rh4*. Short visual fibers (svfs) R1–R6 homogeneously express *rh1* (Salcedo et al. 1999). **b** In *Apis mellifera,* opsin expression in the two lvfs determines three main ommatidia types. In type I, one lvf expresses UV sensitive (light purple), the other blue (blue) sensitive opsin. Both lvfs express UV sensitive opsin in type II, and blue sensitive opsin in type III ommatidia. Short visual fibers uniformly express green sensitive opsin in all ommatidia. The sensitivity and function of the small R9 is unknown (Wakakuwa et al. 2005). **c** In *Papilio xuthus,* UV and blue sensitive opsin expression in the lvfs of type I–III ommatidia is similar as in bees. In all ommatidia types, two svfs co-express two long wavelength-sensitive opsins providing them with maximum sensitivity to green light. The remaining four svfs express red sensitive opsin in type I, red plus green sensitive opsins in type II, and green sensitive opsin in III. Furthermore, spectral sensitivity of *Papilio* photoreceptors is modulated by red (type I and II) or yellow (type III) perirhabdomal pigments and ‘fluorescence pigment’ (type II) (Arikawa 2017). The sensitivity of small R9 is unknown. In the neuronal superposition eye of *Drosophila,* the individual rhabdomeres (gray in a) are spatially and optically separated. In contrast, bee and butterfly ommatidia have a so-called fused rhabdom, where the light-sensitive structures of the individual photoreceptors are grouped closely together and acts as a light guide

*Drosophila*, as most other insects, possesses two compound eyes, each equipped with 750–800 ommatidia. In other insect species, the number of ommatidia per eye can be as low as a dozen and up to several thousand (Lunau 2014). Each individual ommatidium of *Drosophila* houses eight photoreceptors, which is common to Diptera and most other insects. Specific combinations of photoreceptor types give rise to different types of ommatidia. *Drosophila* ommatidia house six outer photoreceptors R1–R6 (short visual fibers, svfs) that project to the first optic neuropil, the lamina, and a pair of superimposed inner R7 and R8 photoreceptors (long visual fibers, lvfs) that project to the second optic neuropil, the medulla (Fig. 3a). Gene regulatory networks and a mix of local signaling events and stochastic mechanisms specify photoreceptor cell fate and give rise to the three ommatidial types of the main part of the *Drosophila* eye (reviewed in Johnston 2013; Mikeladze-Dvali et al. 2005). The large majority of ommatidia belong to the yellow (y) and pale (p) subtype that are randomly distributed at a ratio of roughly 2:1 over the retina (Franceschini et al. 1981; Chou et al. 1996). In *Drosophila*, ommatidia usually follow the ‘one photoreceptor–one rhodopsin’ rule and rhodopsin expression in R7/R8 is tightly coupled: R7p and R8p express *rh3* and *rh5* with maximum sensitivities in the short-UV and blue spectral range, respectively. R7y and R8y express *rh4* and *rh6* with maximum sensitivity in the long-UV and green spectral range, respectively (Salcedo et al. 1999; Figs. 2e,3a). Yellow ommatidia in the dorsally oriented third of the retina (dorsal-yellow, dy) break with the ‘one photoreceptor—one rhodopsin’ rule (Mazzoni et al. 2008). In dy ommatidia, R8 express *rh6* as in y ommatidia, but R7 co-express *rh4* and *rh3* (Fig. 3a). In addition to the three types of ommatidia in *Drosophila*, there are ommatidia specialized in the detection of polarized light in the dorsal-most retina, the so-called dorsal rim area (DRA), that are also present in many other insects (reviewed in Mathejczyk and Wernet 2017). Also in honey bees and *Papilio xuthus*, different combinations of photoreceptors establish three types of ommatidia (Fig. 3b, c). Notably, it has been shown recently that two butterfly species employ gene regulatory mechanisms that resemble mechanisms in *Drosophila* to specify the random ommatidia mosaik (Wernet et al. 2006; Perry et al. 2016).

**Fig. 4:**
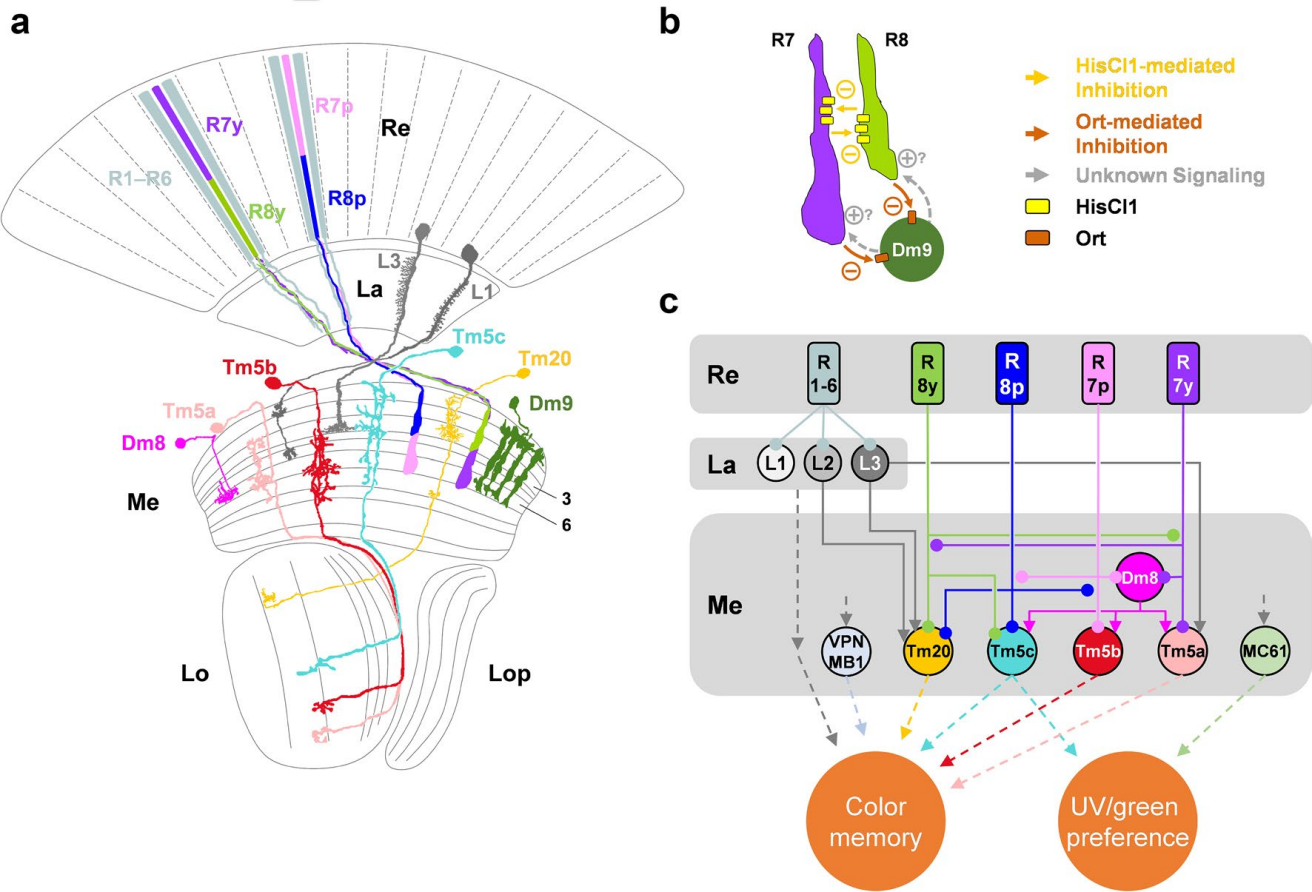
Neuronal basis of color vision in *Drosophila*. **a** Anatomical representation of demonstrated and candidate color processing neurons in the fly optic lobe. Retina (Re), lamina (La), medulla (Me), lobula (Lo), and lobula plate (Lop). **b** R7 and R8 photoreceptors of the same type of ommatidia mutually inhibit each other directly via HisCl1 histamine receptors and receive additional feedback inhibition via Dm9 that requires the second histamine receptor Ort (Schnaitmann et al. 2018; manuscript in preparation). **c** Connectivity and suggested function of the cells in (**a**). The axons of R7 and R8 pass through the lamina and convey information to the distal layers m1–m6 of the medulla. Transmedulla neurons Tm5a, b, c and Tm20, but also amacrine cells including Dm8 receive direct input from R7 or R8. R7 and R8 terminals mutually inhibit each other [see (**b**)]. The svfs of the outer photoreceptors R1–R6 convey information to the lamina monopolar cells L1–L3 that in turn project to the medulla. Simultaneous block of L1 and L2 prohibited blue–green discrimination in a memory task (Schnaitmann et al. 2013). L1–L3 connect to a range of different Tms, among them some with a function in color vision (Tm20 for L2 and L3, Tm5a for L3) (Gao et al. 2008; Takemura et al. 2015). Tm5a,b,c, and Tm20 establish redundant channels of the color vision system (Melnattur et al. 2014). The R7 → Dm8 → Tm5c pathway and the medulla columnar neuron MC61 are necessary for UV/green preference behavior (Gao et al. 2008; Otsuna et al. 2014; Karuppudurai et al. 2014). Tms relay information to the Lobula, for instance, to the lobula intrinsic neuron Li4 and the visual projection neuron LT11 (not shown, Otsuna et al. 2014; Lin et al. 2016). VPN–MB1 establish a direct link from the medulla to the mushroom body, and are necessary for color discrimination in a memory task (Vogt et al. 2016). Round endings and arrowheads denote inhibitory (histamine) and excitatory connections, respectively. Dashed lines indicate unspecified connectivity. Image in (**a**) after Fischbach and Dittrich (1989)

In contrast to bees and butterflies that have a so-called fused rhabdom in which the light-sensitive rhabdomeres are in close proximity and act as a single light guide with a shared optical axis, the rhabdomeres of *Drosophila’s* R1–R6 stay optically isolated from each other (Fig. 3). Only the rhabdomeres of the central photoreceptors R7 and R8 form an optical unit. In this ‘light guide’, R7 filters the light before the remaining light reaches R8 (Trujillo-Cenóz and Melamed 1966; Braitenberg 1967). Furthermore, in Diptera and few other insects (Lunau 2014), one outer photoreceptor of each of six neighboring ommatidia and one pair of R7/R8 from a seventh ommatidium receive light from the same location in visual space. Their axons converge in 1 of about 750 cartridges of the lamina, visual sampling units that reflect both the ommatidial organization of the compound eye and the wiring of ‘neuronal superposition’ eyes (Kirschfeld 1973; Langen et al. 2015). Other than in butterflies and bees, where synaptic interactions in the lamina are likely involved in color opponent processing or in increasing wavelength specificity (Takemura et al. 2005; Chen et al. 2013), lvfs in *Drosophila* pass the lamina without making synapses (Fig. 4, Chen et al. 2019; Meinertzhagen and O’Neil 1991; Menzel and Backhaus 1991; Menzel and Blakers 1976; Ribi 1981; Takemura and Arikawa 2006). The terminals of R7 and R8 in *Drosophila* are often described to ‘precisely’ terminate in medulla layer m6 and m3, respectively (Fig. 4a; Takemura et al. 2008), but reconstructions based on serial electron microscopic data show that R7 forms chemical synapses in m6 and all distal layers, and R8 in m1–m3 (Takemura et al. 2013).

In each lamina cartridge, the signals from R1–R6 are conveyed to the large lamina monopolar cells L1–L3 and to a lamina intrinsic amacrine cell (Meinertzhagen and O’Neil 1991). Eleven different cell types of each cartridge connect the lamina with the medulla, the lamina monopolar cells L1–L5 that send their axons to the medulla, the centrifugal cells C2 and C3 that send their axons to the lamina, T1, Lawf1, and Lawf2 cells (reviewed in Borst et al. 2010; Tuthill et al. 2013). The medulla is the first optic neuropil that receives signals from photoreceptors with different spectral sensitivities and it harbors color vision circuitries (see ‘circuit mechanisms’). In addition to the retinotopic projections of the lamina neurons and the terminals of the lvfs R7 and R8, each medulla column (visual sampling unit of the medulla) houses more than 60 different cell types (Fischbach and Dittrich 1989; Takemura et al. 2013, 2015). Among these are local interneurons including multi-columnar distal medulla (Dm) cell types that laterally connect medulla columns, and transmedulla (Tm) neurons that connect the medulla to the lobula or the central brain. Different cell types directly postsynaptic to R7 and R8 have been identified by genetic labeling techniques and serial electron microscopic reconstructions (Fig. 4a) (Gao et al. 2008; Takemura et al. 2013; Karuppudurai et al. 2014; Takemura et al. 2015). Insights into the contribution of some of these cell types to neuronal circuitries underlying behavior guided by spectral cues are described in detail in our section on circuit mechanism (below). The importance of the medulla and lobula for color vision in insects is generally supported by the observation of color opponent neurons in theses neuropils in different bees and butterflies (Swihart 1972; Kien and Menzel 1977a, b; Hertel 1980; Yang et al. 2004; Paulk et al. 2008, 2009a). The circuitries of the lobula plate have so far not been linked to spectral processing. They play an important role in the computation of visual motion and navigation (see review by Borst et al. 2019 in this issue).

## Behavior guided by spectral cues

In *Drosophila*, dual choice assays were employed to analyze the innate preference of walking flies for monochromatic light against darkness (‘phototaxis’), and to analyze how flies choose between two spectrally distinct lights (‘differential phototaxis’ or ‘spectral preference’). Both paradigms revealed positive phototaxis from 250 to 650 nm with two maxima, one in the blue and one in the UV wavelength range (Bertholf 1932; Fingerman and Brown 1952; Schümperli 1973; Harris et al. 1976; Hu and Stark 1977). When testing phototaxis to UV light of increasing intensity, *Drosophila* preference increases until it saturates at medium intensities. With green light, preference similarly increases up to medium intensities, but decreases and even turns into avoidance at higher intensities (Jacob et al. 1977; Fischbach 1979; Gao et al. 2008). Thus, in *Drosophila*, phototactic behavior is not purely achromatic as it does not exclusively correlate with the intensity of light. Instead, it also depends on the wavelength of presented light and can therefore not be the result of a simple summation of photoreceptor responses. A dependence of phototaxis on the spectral composition of light has further been observed when flies could choose between UV and a mixture of green and UV light (same intensity of UV in both stimuli, i.e. pure UV stimulus in all experiments is darker than the mixture). In these experiments, flies preferred the spectrally mixed stimulus at low UV intensities and the pure UV stimulus at high UV intensities (Heisenberg and Buchner 1977; Fischbach 1979). Altogether, phototaxis in *Drosophila* involves both chromatic and achromatic discrimination. Differentiating between the two in spectral preference experiments with monochromatic stimuli has hitherto not been feasible.

The ecological function of the observed phototactic behavior of *Drosophila* is not known, it may serve orientation behavior towards open space. This assumption largely rests on fact that UV is typically more prominent in the open sky compared with UV reflectance from objects in the ventral half of the field of view (Möller 2002). In line with this assumption, female *Drosophila* orient towards UV light when there is no demand to lay eggs. However, to lay eggs, flies turn away from UV light to lay them on dark substrate (Zhu et al. 2014; Guntur et al. 2017). Thus, phototaxis can depend, at least in mated females, on the internal state. A recent study furthermore suggests that phototaxis in addition depends on the time of the day (Lazopulo et al. 2019). This was observed when analyzing spectral preference of *Drosophila* between blue, red, and green illuminated areas using light intensity levels much higher than in all previous studies. Under these conditions, *Drosophila* showed a strong preference for green in the early morning and late afternoon, reduced preference for green at midday and robust avoidance of blue throughout the day (Lazopulo et al. 2019). It is still somewhat unclear whether these two behaviors (UV avoidance in the context of egg laying and the change of spectral preference during the course of the day) depend on the analysis of spectral content. In honey bees, phototaxis solely depends on the intensity of light (Kaiser et al. 1977; Menzel and Greggers 1985) (but see discussion below). In contrast, many innate behaviors that involve color vision have been reported in insects (Kelber et al. 2003). For instance, the butterflies *Papilio aegeus* and *Pieris brassicae* prefer to oviposit on green substrate, the hoverfly *Eristalis tenax* prefers to feed on small yellow objects and the ant *Camponotus blandus* exhibits a strong innate preference for UV over blue and green stimuli (Lunau and Wacht 1994; Kelber 2001; Yilmaz et al. 2017).

Color vision in *Drosophila* has also been studied using color conditioning paradigms in which the animals were trained to associate a color with either reward or punishment (Menne and Spatz 1977; Kelber et al. 2003; Tang and Guo 2001; Schnaitmann et al. 2010, 2013; Melnattur et al. 2014). In differential conditioning, one spectral stimulus is presented together with, for instance, sugar reward, while another spectral stimulus is presented without. After several rounds of training, flies can choose between the two color stimuli. If flies show a preference for the previously rewarded stimulus, one can infer that they can discriminate the two visual stimuli. Compared with spectral preference experiments, color conditioning greatly facilitates the analysis of intensity invariant stimulus discrimination in *Drosophila*. Changing or reversing the relative intensities of the visual stimuli between training and test in differential conditioning experiments allows to test whether the flies’ choices are based on spectral properties or the intensity of the visual stimuli (Menne and Spatz 1977; Tang and Guo 2001; Schnaitmann et al. 2013; Melnattur et al. 2014). Using differential color conditioning, a wavelength discrimination function (Δ*λ*/*λ*) was determined for *Drosophila* with two wavelength ranges where the capability to discriminate neighboring wavelengths is highest. In the violet (≈ 420 nm) and in the blue–green (≈ 495 nm) wavelength range, flies can discriminate stimuli that differ about 20 nm in wavelength (Hernández de Salomon and Spatz 1983). In their important pioneering study, Hernández de Salomon and Spatz (1983) tested only relatively few wavelengths and the missing analysis of UV excludes major sensitivities of the *Drosophila* compound eye (Fig. 2e). In addition, they used spectral preference behavior to reveal isoluminant stimuli. This approach is questionable because spectral preference itself depends on both spectral and intensity properties of stimuli. Thus, a potential contribution of intensity discrimination cannot be excluded. When compared with bees or butterflies, the obtained Δ*λ*/*λ* values for *Drosophila* are rather large. Differential color conditioning experiments in freely flying honey bees revealed a Δ*λ*/*λ* function with two optima, one in the violet (≈ 400 nm) and one in the blue–green (≈ 500 nm) wavelength range. However, bees were able to discriminate stimuli differing only 4–6 nm in wavelength (von Helversen 1972). An even more accurate and complex color discriminability was found in the butterfly *Papilio xuthus.* Using proboscis extension reflex (PER) conditioning (Koshitaka et al. 2008), the Δ*λ*/*λ* function exhibits three optima at 420, 480 and 560 nm, respectively, and butterflies were able to discriminate stimuli as close as 1 nm apart from each other.

Importantly, color discrimination in *Drosophila* strongly depends on the precise design of the behavioral assay, and whether flies are agitated or undisturbed prior to behavioral decisions (Harris et al. 1976; Heisenberg and Buchner 1977; Jacob et al. 1977; Fischbach 1979; Gao et al. 2008). Dependencies on the behavioral assay were also observed in bees. Investigation of wavelength discrimination using either the proboscis extension reflex in restrained bees or behavioral decisions in freely moving bees as readout revealed much better wavelength discrimination in the latter (Niggebrügge et al. 2009). Also, differential conditioning yields better discrimination than absolute conditioning in bees (Giurfa 2004). If spectral discrimination in *Drosophila* also depends on the angular size of the presented stimuli remains unknown. In bees, discrimination relies exclusively on achromatic L-receptor contrast if stimuli are small (5°–15° of visual angle), whereas larger stimuli are discriminated exclusively based on chromatic contrast (Giurfa et al. 1996, 1997; Giurfa and Vorobyev 1998; Ng et al. 2018). Thus, more than ~ 60 neighboring ommatidia must be stimulated to enable object detection based on chromatic mechanisms in bees. In contrast, stimulation of only seven ommatidia enables object detection based on L-receptor contrast. These findings might also provide a simple explanation for experiments in which bee phototaxis behavior was found to be achromatic: the presented stimuli of 1–9.5° of angular size might have been too small to enable chromatic processing mechanisms to become active (Kaiser et al. 1977; Menzel and Greggers 1985). With respect to color vision research in *Drosophila*, it is important to note that spatial color contrast and color constancy, that are well documented in at least some insects including butterflies and bees have not been reported so far (reviewed in Chittka et al. 2014). In contrast, temporal color contrast is detected by *Drosophila* (Fischbach 1979).

## Photoreceptor contributions to color vision

In the 1960s, Seymour Benzer pioneered the field of neurogenetics by isolating *Drosophila* mutants with deficits in phototaxis using chemical mutagenesis (Benzer 1967). Later, further mutants with impaired vision were isolated based on noticeable defects in electroretinogram recordings (Koenig and Merriam 1977). Specific structural and functional defects were assigned to in particular the mutants *rdgB*^*KS222*^, *sev*^*LY3*^ and ora^JK84^ that were used to reveal the contribution of photoreceptors to phototaxis and spectral preference (Harris et al. 1976; Heisenberg and Buchner 1977; Hu and Stark 1977; Jacob et al. 1977; Fischbach 1979). In ora^JK84^ and *rdgB*^*KS22*^, R1–R6 completely degenerated in the presence of light. In *sev*^*LY3*^, mutant R7 photoreceptors are completely absent due to a developmental defect. Studies based on these mutants suggested that all photoreceptors can contribute to phototaxis and spectral preference. However, it became clear that their contributions critically depend on the exact stimulus conditions. Spectral preference behavior to two monochromatic stimuli, with one as a constant reference and the other varied in intensity and wavelength, is mainly mediated by R7 and R8 (Hu and Stark 1977). In contrast, when spectral preference is tested with a ‘white’ reference stimulus, it involves all photoreceptor types (Schümperli 1973; Harris et al. 1976). Phototaxis experiments also revealed that R1–R6 have a higher absolute light sensitivity than R7/R8 (Harris et al. 1976; Jacob et al. 1977).

Years later, it was shown that *sev*^*LY3*^ causes uniform *rh6* expression in all R8 photoreceptors in addition to the known R7 degeneration (Chou et al. 1999). *ora*^*JK84*^ turned out to be a double mutant with null mutations in *ninaE* (*rh1*) and *ort* (O’tousa et al. 1989). The previously unnoticed mutation in *ort* affects one of two histamine receptor genes of the *Drosophila* genome that is expressed widely in the fly visual system in all neurons downstream of all types of photoreceptors (Gao et al. 2008). Thus, the *ort* mutation included in *ora*^*JK84*^ strongly affects visual processing, in addition to the known absence of R1–R6 function. More recent studies that employed either specific mutations in individual rhodopsin genes or specific rhodopsin promoters to drive block of synaptic transmission in specific photoreceptor types overcome these problems. However, despite significant differences in the specificity of the approaches, the new studies largely corroborated the major findings of older work and demonstrated that each photoreceptor type (R1–R6, R7, R8) can drive spectral preference on its own (Gao et al. 2008; Yamaguchi et al. 2010).

Few studies addressed whether individual photoreceptor classes contribute to the chromatic aspect of phototactic behavior. Color mixing experiments revealed that *sev* mutants did not exhibit the wavelength dependent reactions to mixed UV/green stimuli that were exhibited by wild-type flies (see ‘behavior guided by spectral cues’, Heisenberg and Buchner 1977; Fischbach 1979). Furthermore, *sev* mutant flies did not show the negative phototaxis at high intensities of green light (Jacob et al. 1977). These findings suggest that R7 has a prominent role in the chromatic aspect of phototactic behavior.

Based on their broad-band sensitivity and major role in motion vision (Heisenberg and Buchner 1977; Yamaguchi et al. 2008; Wardill et al. 2012), R1–R6 were thought not to contribute to fly color vision (Troje 1993). This view was challenged by the finding that *Drosophila* color discrimination is described best using a receptor noise-limited model that incorporates R1–R6 (Hernández de Salomon and Spatz 1983; Schnaitmann et al. 2013; Garbers and Wachtler 2016). Most importantly, flies with R1–R6 and R7y as only functional photoreceptors are able to discriminate blue and green (Schnaitmann et al. 2013). This finding rests on experiments combining genetic interference with photoreceptor function and appetitive color conditioning. However, it turned out that R1–R6 are not required in general for this specific spectral discrimination: flies with function restricted to R7y and R8y were also able to discriminate blue and green, whereas flies with function restricted to R1–R6 and R8y failed. These experiments suggest that fly color vision involves comparisons between R1–R6 and R7y as well as R7y and R8y. This conclusion is in line with the involvement of R7 in the chromatic aspect of phototaxis (see above). Flies with function restricted to R1–R6, R7p and R8p were not able to discriminate blue and green. To further explore the role of photoreceptor types in *Drosophila* color vision, the range of the wavelengths that are analyzed in spectral discrimination tasks must be extended. In particular, it remains to be addressed whether also the photoreceptors of pale ommatidia are involved in the discrimination of shorter wavelengths, as suggested by work in *Lucilia* (Troje 1993).

Color discrimination in bees involves all photoreceptor types, and computational models including all three photoreceptor types explain behavioral observations well (Neumeyer 1980; Backhaus et al. 1987; Backhaus 1991; Chittka 1992; Brandt and Vorobyev 1997; Vorobyev et al. 2001). Electrophysiological recordings in the bee visual system revealed a large diversity of different color opponent response types, and modeling work demonstrates that all three photoreceptor types are involved (Kien and Menzel 1977a, b; Hertel 1980; Yang et al. 2004; Paulk et al. 2008, 2009a, b; Vasas et al. 2019). In contrast, color discrimination in *Papilio xuthus* does not involve all of its six ‘spectral classes’ of photoreceptors (Koshitaka et al. 2008). The Δ*λ*/*λ* function of these butterflies exhibits three optima that are interpreted as evidence for three photoreceptor opponent computations in color vision. A receptor noise-limited color opponency model including UV, blue, green, and red photoreceptor sensitivities yields the best fit to the Δ*λ*/*λ* function (Koshitaka et al. 2008; Arikawa 2017).

## Circuit mechanisms underlying color vision

Physiological studies that include detailed investigations of the identity, function and nature of synaptic and circuit interactions in insect color vision are still rare. Until recently, this included the physiology of insect photoreceptor terminals that were uncharted territory (see below). Intracellular recordings from distal segments of photoreceptors of many insect species showed positive membrane potential changes; only few studies reported additional evidence for spectral inhibition. Seldom encounters of additional negative interactions have been reported in bees where some of the recorded UV receptors depolarized at short wavelengths and hyperpolarized at wavelengths longer than 420 nm. Comparable hyperpolarizing responses have been observed in some UV- and B-receptors in bumble bees (Skorupski et al. 2007). Why such hyperpolarizing responses were only observed in few of the recordings remained unknown. Most photoreceptors were excited over a wavelength range broader than expected from rhodopsin sensitivity (Menzel and Blakers 1976). Moreover, the spectral inhibition was absent when recordings were made in axons in the lamina instead of outer photoreceptor segments. These axonal recordings revealed ‘pure single pigment spectral sensitivity’, which led the authors to conclude that positive and negative interactions cancel each other (Menzel and Blakers 1976).

Clear evidence for inhibitory photoreceptor interactions in insects comes from experiments in few butterfly species where a fraction of the recorded photoreceptors exhibited hyperpolarizing responses to light (Horridge et al. 1983; Matić 1983; Chen et al. 2013). In these intracellular recordings, the reference electrode was usually placed in the body cavity and Matić (1983) showed that many of the hyperpolarizing interactions were strongly reduced or turned into excitation when the reference electrode was placed in the extracellular space close to the recording electrode. He explained this observation by the presence of strong light-induced fluctuations of the extracellular potential of up to 60 mV (Matić 1983). However, because inhibition was retained in some of the recordings, Matić concluded that inhibition between photoreceptor is not a methodological artifact and that butterflies make a special case.

New insights came from a recent study in *Drosophila.* Two-photon calcium imaging with subcellular resolution revealed UV_short_ vs. blue and UV_long_ vs. green opponent processing in the terminals of R7p/R8p and R7y/R8y, respectively (Schnaitmann et al. 2018). These first color opponent interactions in the fly visual system rely on mutual inhibition between R7 and R8 of either type of ommatidia. Notably, this spectral inhibition is mediated by two mechanisms that act in parallel. First, the *Drosophila* histamine-gated chloride channel HisCl1 is expressed in inner photoreceptor terminals and mediates direct reciprocal synaptic inhibition between R7 and R8 of the same ommatidium (mutual inhibition within a single column). Second, R7 and R8 receive spectral feedback signals from neurons in the medulla that express the second *Drosophila* histamine receptor Ort, in particular from the multi-columnar Dm9 cell as revealed in recent work of our laboratory (Fig. 4b) (Schnaitmann et al. 2018; manuscript in preparation). These two mechanisms are of different importance in R7 and R8: in R7, either of the mechanisms is sufficient for color opponent processing, whereas in R8 both mechanisms are required and must act together for detectable color opponency (Schnaitmann et al. 2018). Inhibitory photoreceptor responses, synaptic contacts between photoreceptors, and expression of PxHCLB, the homolog of *Drosophila* HisCl1in *Papilio xuthus* suggest that related neuronal mechanisms underlie early color opponent processing in butterflies and flies (Takemura and Arikawa 2006; Takemura et al. 2013; Schnaitmann et al. 2018; Chen et al. 2019). Differences between *Drosophila* and butterflies exist in the precise site where interactions take place. In *Drosophila*, HisCl1-mediated photoreceptor interactions are restricted to the distal three layers m1–m3 of the medulla (Schnaitmann et al. 2018). In *Papilio xuthus*, the major site of PxHCLB expression is the lamina with additional neuronal expression in the medulla. Furthermore, vfs as well as svfs express the histamine receptor PxHCLB and inter-photoreceptor connectivity is more complex than in *Drosophila* (Chen et al. 2019).

The pale and yellow specific spectrally opponent processing mechanisms in *Drosophila’s* R7/R8 terminals match the opponencies that were suggested to underlie color discrimination in *Lucilia* (Troje 1993), and are in line with the finding that R7y/R8y are sufficient for blue–green discrimination in *Drosophila* (Schnaitmann et al. 2013) (see above). Together, these findings suggest that color vision in *Drosophila* involves UV_short_/blue and UV_long_/green color opponent pathways and that these pathways emerge already at the photoreceptor level. Thereby, processing in inner photoreceptor terminals very likely is the first of a series of sequential color opponent processing steps that remain to be revealed. Further color opponent processing steps in higher-order visual neurons are also suggested when the results from two-photon calcium imaging and behavioral experiments are compared. Functional imaging revealed no evidence for the integration of R1–R6 signals at the level of R7/R8 terminal responses (Schnaitmann et al. 2018). However, a contribution of R1–R6 and of their downstream neurons L1 and L2 to color discrimination has been demonstrated in a behavioral study (Schnaitmann et al. 2013; Garbers and Wachtler 2016). Therefore, the signals of R1–R6 must be integrated into the *Drosophila* color vision circuitry downstream of R7/R8 terminals. Combined genetic, anatomical, and behavioral studies suggest that R1–R6 signals are conveyed via L2 and/or L3 to Tm5 subtypes and Tm20, neurons that are directly downstream of R7/R8 (see below and Fig. 4). Whether these neurons indeed generate color opponent responses that are in line with indirect input from R1–R6 remains to be revealed by physiological recording.

To this date, the function of neurons downstream of R7/R8 in color vision has been addressed by combining promoter- or cell-type-specific genetic targeting with anatomical characterization, connectivity analysis, functional perturbation, and behavioral analysis (Gao et al. 2008; Karuppudurai et al. 2014; Melnattur et al. 2014). In a pioneering study, Gao et al. (2008) identified neurons that are postsynaptic to R7/R8, including several candidate neurons of the color vision circuitry. Because arthropod photoreceptors release histamine as sole neurotransmitter (Hardie 1989; Stuart 1999), the authors reasoned that direct synaptic interactions with second-order interneurons should be mediated by the inhibitory histamine-gated chloride channel Ort that is widely expressed in the lamina and medulla of the fly visual system (Witte et al. 2002; Pantazis et al. 2008). Comparison of *ort*-promoter activity with the stratification of axonal endings of R7/R8 revealed candidate neurons postsynaptic to R7/R8. Subsequently, synaptic connectivity was thoroughly confirmed using genetic labeling techniques (GRASP or derivatives thereof) and serial electron microscopic reconstructions (Gao et al. 2008; Takemura et al. 2013; Karuppudurai et al. 2014; Takemura et al. 2015). This resulted in the identification of the transmedulla projection neurons Tm5a/b/c, Tm9 and Tm20, and the multi-columnar amacrine neuron Dm8 (Fig. 4). Tm5a/b/c, Tm9, and Tm20 receive direct and/or indirect input from inner photoreceptors. Some of them additionally receive indirect input from R1–R6 via the lamina monopolar cells L2 and/or L3 (Takemura et al. 2013, 2015). Of these neurons Tm9 was later associated with motion vision, as it conveys information to T5 and is therefore part of the OFF-edge motion detection pathway (Shinomiya et al. 2014; Serbe et al. 2016).

Dm8 and Tm5c neurons have a central function in UV/green spectral preference. Dm8 is postsynaptic to R7, has multi-columnar organization, and expresses Ort. Individual Dm8 cells pool ‘UV input’ from 12 to 16 R7 photoreceptors of neighboring ommatidia and conveys it to all Tm5 subtypes, as well as unknown neurons (Gao et al. 2008; Karuppudurai et al. 2014; Menon et al. 2019). Blocking synaptic output of glutamatergic Dm8 results in reduced preference for UV, whereas flies with restored Ort receptor function in exclusively Dm8 (cell specific Dm8 rescue in *ort* mutant flies) exhibit intact spectral preference behavior (Gao et al. 2008). Knock-down of kainate glutamate receptors in postsynaptic Tm5c reduces preference to UV, suggesting that direct input from Dm8 to Tm5c is important. In line, flies with blocked synaptic transmission in Tm5c show defects in spectral preference comparable to flies with blocked synaptic transmission in Dm8 (Karuppudurai et al. 2014). Furthermore, Ort expressing Tm5c receive direct synaptic input from R8 and this input contributes to preference for green. Altogether, the R7–Dm8–Tm5c pathway is necessary and sufficient to mediate UV spectral preference against green light.

In addition to its role in UV/green spectral preference behavior, Tm5c together with other Tm cell types contributes to blue/green discrimination in a conditioning assay (Melnattur et al. 2014). Simultaneous block of Tm5a/b/c, and Tm20, but not of single cell types or combinations of them is required for complete loss of blue–green color discrimination (Melnattur et al. 2014). Therefore, Tm5a/b/c, and Tm20 are thought to represent redundant pathways of the color vision system. These neurons convey jet unknown information to visual projection neurons in the deep strata of the lobula that project to the central brain (Otsuna and Ito 2006; Lin et al. 2016; Panser et al. 2016; Wu et al. 2016). The lobula intrinsic neuron Li4 makes many synaptic contacts with almost all Tm5a/b/c and Tm20. LT11 is less connected with most of the Tms, apart from extensive Tm5a input (Lin et al. 2016). Blocking synaptic transmission in LT11, a single neuron per hemisphere that receives input from the entire visual field and therefore cannot convey retinotopic information, caused defective phototaxis specifically in the range of 410–440 nm (Otsuna et al. 2014). The same study identified another type of visual projection neuron, MC61, that projects from the medulla to the anterior optic tubercle, bypassing the lobula. Blocking its synaptic output caused a phototaxis defect in the green and UV wavelength range (Otsuna et al. 2014). Also, recent research on associative visual information processing in *Drosophila* revealed direct pathways from the medulla to the central brain (Vogt et al. 2014, 2016). These VPNs project to the mushroom body, a prominent brain structure involved in associative memory. Thereby, different subpopulations of VPN are required for memories of color (VPN–MB1) and brightness (VPN–MB2) (Vogt et al. 2014, 2016).

## Outlook

At present, it is unknown whether or how the p–y dichotomy that has been observed in R7/R8 presynaptic terminals is retained in the pathways of the *Drosophila* color vision system (Schnaitmann et al. 2018). Anatomical data show that Tm5a, which is found exclusively in y columns, receives input from R7y but not R7p, and that Tm5b receives predominately input from R7p (Fig. 4c) (Karuppudurai et al. 2014; Menon et al. 2019). In contrast, Tm5c appears to pool p and y inputs (Fig. 4c). Recently, genetic and molecular methods enabled new insights into the specification of Dm8 cells. Analysis of the expression of the surface molecules of the Dpr and DIP family suggests that different flavors of Dm8 exist that likely correspond with p and y subtypes. Dpr11 is selectively expressed in R7y that contact a subpopulation of Dm8 that in turn express the Dpr11 interaction partner DIP-γ; absence of these interaction partners characterize R7p and another subpopulation of Dm8 (Carrillo et al. 2015; Tan et al. 2015; Menon et al. 2019).

What kind of color-coding physiological response types can be expected in second- and higher-order visual interneurons in *Drosophila*? To speculate on this question, a look at color vision in bees may provide important suggestions. Early studies on bee color vision suggested a small number of color opponencies that are likely established by deterministic wiring of the three types of photoreceptors to postsynaptic neurons. However, electrophysiological examination so far revealed a large diversity of color opponent responses with different spectral tuning (Menzel and Blakers 1976; Kien and Menzel 1977a, b; Hertel 1980; Yang et al. 2004; Paulk et al. 2008, 2009a, b). This unexpected diversity might be explained by partially random wiring of photoreceptors to postsynaptic color opponent neurons in the medulla and lobula (Vasas et al. 2019). It would also help to explain why receptive fields with a spatially antagonistic organization have not been reported so far in bees (Kien and Menzel 1977a, b; Hertel 1980). In *Drosophila*, it is now time to reveal the diversity of color opponent neural cell types in the medulla and lobula, the spatial layout of their receptive fields, the underlying cellular wiring, and the molecular implementation of identified computations. We expect this research to provide conceptually new insights into the neuronal basis of color vision in insects and important visual phenomena like spatial and temporal color contrast and color constancy. The recent connectomics and RNAseq data of the medulla and its cell types will fuel this research (Takemura et al. 2013, 2015; Tan et al. 2015; Konstantinides et al. 2018).
